# Isolation, morphological characterization, and screening a virulence of *Beauveria bassiana *and *Metarhizium* *robertsii *as bioagents

**DOI:** 10.12688/f1000research.134020.3

**Published:** 2023-10-30

**Authors:** Dereje Geremew, Tadale Shiberu, Ararsa Leta

**Affiliations:** 1 Ethiopian Institute of Agricultural Research,Ambo Agriculture Research Center, Ambo, Ethiopia; 2 Ambo University, Ambo, Oromia, 1000, Ethiopia

**Keywords:** Conidia, colony color, endophytic, entomopathogenic fungi, Galleria melonella, isolates

## Abstract

**Background:** Entomopathogenic fungi exists naturally in plants as an asymptote and has the potential to reduce the population of insect pests through indirect interactions. This study was conducted to detect and characterize the endophytic fungi *Beauveria bassiana* and *Metarhizium robertsii* from the rhizosphere soil of tomato plants and their virulence effect on *Galleria melonella.*

**Methods:** From the rhizosphere soil of 40 tomato fields, three *Beauveria bassiana* and seven *Metarhizium robertsii* isolates were identified using the galleria bait method. All fungi isolates were morphologically characterized by their colony color, shape, and surface texture. Isolates with the highest percentages of germination, conidial yield, and radial growth were selected, and their pathogenicity was evaluated on second instar larvae of *Galleria melonella* under laboratory conditions.

**Results:** In this study, *Beauveria bassiana* showed white colony color and aseptate hyphae, whereas *Metarhizium robertsii showed dark green to light green colony color and septate hyphal structures.* Maximum spore production and conidial length were obtained by *Beauveria bassiana* isolate APPRC-27 with 2.67x10 ^7^ spores ml ^-1^ and 3.24 µm, respectively. Colony radial growth rates ranged from 1.73 to 3.24 mm day ^-1^. The results revealed that the highest mortality rate of *Galleria melonella* (100%) was obtained by *Metarhizium robertsii* isolates K-61 and K-102 at a concentration of 1x10 ^8^ conidial ml ^-1^ at 7 days post-inoculation. The lowest mortality rate was registered by *Metarhizium robertsii* isolate RST-11.

**Conclusions:** In the present study, isolates *that produced the most spores and had the highest germination rates were the most virulent to Galleria mellonella second instar larvae.* Therefore, virulent entomopathogenic fungi, *Beauveria bassiana* and *Metarhizium robertsii*, are promising bioagents for the control of pests.

## Introduction

Entomopathogenic fungi, *Beauveria bassiana*, (Bals. -Criv.) Vuill. and *Metarhizium robertsii* (Metchnikoff) Sorokin, live in plant tissues without showing pathological symptoms ( Dutta *et al*., 2014). They have the potential to limit the feeding capacity, preference, and oviposition of stem boring, sap-sucking, and leaf mining insects on many crops by producing toxic compounds that disturb the insects performance. Additionally, they increase the plant defense mechanisms against pests or abiotic factors ( McCormick *et al*., 2016; Hokkanen and Menzler-Hokkanen, 2017). Entomopathogenic fungi are capable of penetrating their insect hosts through cuticular exoskeletons ( Zimmermann, 2007).

Entomopathogenic fungi have become more promising than chemical pesticides for the control of pests in Ethiopia due to pesticide resistance. This is due to the misuse of pesticides, which causes the incrementation of pesticide-resistant biotypes ( Negash *et al*., 2020), and a lot of pests have developed resistance due to the high use of pesticides ( Tabashnik *et al.,* 2009). Consequently, using eco-friendly biopesticides like *B. bassiana* and *M. robertsii* as bioagents against agricultural pests is the best option to increase crop production and productivity. They are symbiotic with their hosts, reduce pest populations, and require little inoculum for their systemic transmission within plant tissues ( Nair and Padmavathy, 2014). Moreover, entomopathogenic fungi are the immediate solution for pests that feed inside leaves, stems, roots, and seeds because they reduce their exposure to synthetic insecticides and other control methods ( Resquín-Romero *et al*., 2016).

Identification and characterization of entomopathogenic fungi, *B. bassiana* and *M. robertsii,* are highly required for entomological research and essential for the selection of virulent isolates against several pests ( Boucias *et al*., 2000). Currently, the selection of virulent strains of entomopathogenic fungi as the biological control agent of pests is of great interest to many researchers ( Lacey *et al*., 2015). However, there is a limit to the work on the isolation, morphological characterization, and screening of virulent isolates of entomopathogenic fungi, particularly *B. bassiana* and *M. robertsii* in Ethiopia, to control agricultural pests. Hence, this study was made to identify, characterize the morphology, and screen the virulence of *B.* *bassiana* and *M. robertsii* isolates on *Galleria melonella* collected from rhizosphere soil in tomato plant farms at Koka and Guder areas in Ethiopia.

## Methods

### Description of the study site

The experiment was conducted at the Ambo Agriculture Research Center under laboratory conditions. Ambo is far away from Addis Ababa, 110 km to the west, at a geographical coordinate of 8°59′N latitude and 37.85°E longitude, with an altitude of 2,100 m a.s.l. The daily temperature ranges from 15 to 29°C, with an annual temperature of 22°C on average. The soil sample was collected from two sites, namely Guder and Koka in Ethiopia. Guder is located at a latitude and longitude of 8 ^o^58′N and 374 ^o^6E, respectively, with an altitude of 2,101 m a.s.l., whereas Koka is found at 8 ^o^25′ N latitude, 39 ^o^02′ E longitude, and an altitude of 1,605 m a.s.l. The minimum and maximum temperatures ranged from 12.14 to 27.39°C, respectively ( Etefa and Dibaba, 2011).

### Sampling of rhizosphere soil from tomato plants

Rhizosphere soil was collected from tomato farmlands by uprooting vegetative-stage tomato plants. During the collection, the upper layer of soil was removed. A total of 40 rhizosphere soil samples were taken from two locations. Five samples were taken from each spot and mixed in a plastic bag before being taken to the bio-control laboratory of the Ambo Agriculture Research Center for the isolation of B. *bassiana* and *M. robertsii.* Finally, one kilogram (kg) of the soil sample was used for the isolation by removing unnecessary parts from the soil ( Sevim *et al*., 2010).

### Isolation of *B. bassiana* and *M. robertsii* from Rhizosphere soil samples

From 40 soil samples, *B. bassiana* and *M. robertsii* were isolated by using the Galleria baiting method as follows ( Zimmermann, 1986; Belay *et al*., 2017).

One kilogram of soil sample was moistened by sterilized water and added to a 1.5 kg screw-capped glass jar. A total of 10 wax moth larvae were separately introduced into jars filled with soil samples and incubated at 26°C for 10 consecutive days under dark conditions.

Larval deaths were inspected and recorded every three days. The moisture of the soil was maintained by moistening it with sterile water daily. The cadavers were taken off the soil and surface sterilized with 1% sodium hypochlorite for 2 minutes, followed by 70% ethanol for 10 seconds, and rinsed three times with sterile water. Surface-disinfected larval cadavers were placed on sterile plastic plates with slightly wetted filter paper and incubated at room temperature up to the sporulation of outgrown mycelia.

The sporulation of *B. bassiana* and *M. robertsii* over the dead cadavers was examined, and the spores were harvested by a sterilized inoculating wire loop, transferred onto Sabouraud Dextrose Agar (SDA) Microxpress ^®^ media, and incubated at 27±1°C for 14–21 days. Isolates were purified by subculturing on newly prepared SDA media, and pure cultures were preserved with glycerol and maintained on agar at -19°C for further work.

### Morphological identification and characterization of isolates

Fungal isolates were morphologically identified and characterized using the identification key of Humber ( Humber, 1997), depending on fungal structures acquired from pure culture ( Gürlek *et al.*, 2018). In addition to the new isolates, three B. *bassiana* (APPRC-44BC, APPRC-44BC-1, and APPRC-27) and one *M. robertsii* (S-26) isolates were also characterized from the Ambo Agriculture Research Center as standard checks for the comparison of isolates.

Consequently, the morphologies of B. *bassiana* and *M. robertsii* were characterized by the culturing of pure cultures on the SDA media, and inspection was done for 14–21 days. Both macroscopic and microscopic characterization of isolates was conducted by observation of morphological features of isolates. Macroscopic features such as mycelial color, colony reverse, colony shape and color, colony elevation, and surface texture were observed. Microscopic characterization of isolates was also done under an Olympus SC50 (CX33RTFS2, Olympus Corporation) microscope with ×40 magnification ( Barra *et al.,* 2013) by measuring conidial length and diameter, hyphal growth, and conidiogenous cells using the slide culture technique.

### Slide culture preparation

The technique was conducted by autoclaving a 9-cm-diameter Petri dish with filter paper and a bent glass rod. A 15 g ^-1^ of water agar was cut by a 1 cm × 1 cm block with a sterilized scalpel blade and put on a glass slide. Fungal spores were inoculated on the four sides of the agar blocks by a sterile inoculating needle, and coverslips were placed on the block. Moistened filter paper with 2 ml of sterile water was placed over plates, sealed with parafilm, and incubated at 27±1°C.

Agar blocks were discarded after incubation for 72 hours, and the slides were moistened with a drop of 97% ethanol and enabled to heat fix by passing on an alcohol lump. A drop of lactophenol blue was used to stain the slide with a coverslip, which was later sealed with nail polish. The slide was placed under a SC50 microscope with ×40 magnification for the observation and characterization of conidiophore structure ( Rosana *et al*., 2014). Finally, the slides were permanently preserved for further characterization. The characterization and measurement of spore features such as conidial length and diameter were also done using the Sc 50 microscope (magnification, ×40).

### Spore germination test for the viability of fungal isolates

The spore germination test was verified by the conidial germination test technique according to standard procedures ( Goettel and Inglis, 1997). Fungal spores were harvested from a 21-day-old culture by scraping with a sterilized spatula. Harvested spores were added to 10 ml of sterile water with Tween 80 (0.001% v/v) as a surfactant in a falcon tube and vortexing. Fungal spores were adjusted to 1 × 10 ^6^ conidia ml ^-1^ by using a Neubauer hemocytometer (USA) under a SC50 microscope.

In total, 100-μl of suspended spores was spread over fresh Potato Dextrose Agar (PDA) (Himedia ^®^), and two sterilized glass slides were placed on the media and incubated at 28°C for 18 hours. After 18 hours, germination of the spores was halted by dispensing with 70% ethanol. Both germinated and non-germinated cells were counted by using a Neubauer hemocytometer under a SC50 microscope (magnification, ×40).

The experiment was conducted under laboratory conditions in CRD with 15 treatments and three replications. A germ tube with more than half the diameter of the spore was considered germinated, and *vice versa* for non-germinated. Isolates with more than 95% germination were selected against *G. melonella* to test their pathogenicity and used for further work. The percentage of spore germination was calculated according to the method developed by Vega *et al*. (2008).

$$\text{Percent spore germination}=\frac{\text{number of spores germinated}}{\text{Total spore counted}}\times 100$$

### Radial growth rate

Conidia of 14-day-old cultures were harvested from plates and spread on a 90 mm Petri dish containing fresh SDA and PDA media separately. Isolates were incubated for three days at 27±1°C, and the 5 mm diameter of three-day-old cultures mycelium was taken by using a cork borer and placed in the center of a 90 mm petri dish containing SDA and PDA media individually. Cultures were incubated at 27±1°C ( Membang *et al.,* 2021).

The experiment was conducted in CRD with 11 treatments and replicated three times. Data on radial growth were recorded in mm/day from the 4 ^th^ to the 15 ^th^ days and calculated by:

$$\mathrm{RG}/\mathrm{day}=\frac{\text{Colony diameter at the end of incubation period}-\text{Fungal disc diameter}}{\text{Total incubation days}}$$

Where, RG/day is radial growth per day.

### Estimation of conidial yield

Spore production was estimated as follows ( Schemmer *et al*., 2016). A 21-day-old culture of 10 mm-diameter circular agar was cut with a cork borer, and the disc was added to 10 ml of sterile 0.001% Tween 80 solution. The solution was well vortexed to evenly mix, and the conidial concentration was counted by using a Neubauer hemocytometer. The conidial yield was expressed in terms of conidial ml ^-1^. The experiment was conducted under laboratory conditions in CRD with eight treatments and three replications.

### Conidial suspension preparation

Conidia were obtained from pure cultures grown on SDA, which was incubated for 14 days at 27±1°C under dark conditions. Conidia were harvested by disposable cell scrapers and added into test tubes containing Tween 80 (0.001% v/v). Suspensions were vortexed well for 2 minutes and adjusted to 1 × 10 ^8^ conidia ml ^-1^ using a Neubauer hemocytometer ( Gurulingappa *et al*., 2010).

### Rearing of *Galleria melonella*

*Galleria mellonella* larvae (Lepidoptera: Pyralidae) were reared at the Ambo Agriculture Research Center in the biocontrol room following the methods described by Meyling (2007). Adult moths were kept in 500-ml flasks containing folded tissue paper to fasten their mating and egg-laying capacities. To hatch third- to fourth-instar larvae, the eggs were laid on folded tissue paper, transferred to plastic containers filled with 80 g honey, 50 g wheat bran, and 180 g glycerol, and incubated at 20°C for four weeks in the dark.

### Pathogenicity screening of isolates using *G. mellonella*

The potential virulence of fungal isolates was tested using 2nd instar *G. mellonella* larvae by the galleria dipping method. Spores collected from 14–21 days of old culture by sterilized spatula scraping were adjusted to 1 × 10 ^8^ spores’ ml ^-1^ and 10 ml were prepared from the suspension of sterilized water with Tween 80 (0.001% v/v) in a sterilized falcon tube and vortexing well. A total of 10 second instars of *G. mellonella* larvae were dipped into spore suspensions for 15 sec. Treated larvae with fungal isolates were incubated at 26°C for 10 days under dark conditions. Cadavers were collected every day 3 days post inoculated, disinfected on their surfaces, transferred into sterilized plastic plates lined with moistened filter paper, and incubated in a dark place. Dead larvae were daily inspected and moistened with sterilized water to promote the outgrowth of mycelial on the cadavers. A control was treated with Tween 80 (0.001% v/v) and incubated under the same conditions ( Ayele *et al*., 2020). Isolates with greater than 90% mortality of larvae were selected, considered to be virulent isolates, and preserved for further work.

### Data analysis

Germination test, radial growth, conidial yield and size, and screening of isolates data were analyzed using analysis of variance (ANOVA) followed by Tukey’s test with a statistical difference of P<0.05. Statistical Analysis System (SAS) (RRID:SCR_008567) software 9.4 version was used for data analysis. Means were separated using Tukey’s honestly significant difference (HSD) at P<0.05.

## Results

### Morphological identification and characterization of *B. bassiana* and *M. robertsii*

This study revealed that three *B. bassiana* and seven *M. robertsii* isolates were identified from rhizosphere soil samples of tomato plants. *B. bassiana* isolates showed white colony color, wide round to round colony shape, smooth powdery to smooth cottony surface texture, and raised colony elevation, as indicated in Table 1.

**Table 1.** Macroscopic features of *B. bassiana* and *M. robertsii.*

Isolate name	Isolate code	Source	Location	Host plant	Colony Color		Colony shape	Surface texture	Colony elevation
					front	back			
*B. bassiana*	RST-8	Rh. soil	Guder, Ethiopia	tomato	white	yellowish	wide round	smooth powder	raised
*B. bassiana*	APPRC-27	soil	Ambo ARC collection	unknown	white	yellowish	round	smooth, powder	raised
*B. bassiana*	K-91	Rh. soil	Koka, Ethiopia	tomato	white	yellowish	round	smooth, cottony	raised
*B. bassiana*	K-5	Rh. soil	Koka, Ethiopia	tomato	white	yellowish	round	smooth, cottony	raised
*B. bassiana*	APPRC-44BC	soil	Ambo ARC collection	unknown	white	yellowish	round	smooth, powder	raised
*B. bassiana*	APPRC-44BC-1	soil	Ambo ARC collection	unknown	white	yellowish	round	smooth, powder	raised
*M. robertsii*	RST-11	Rh. soil	Guder, Ethiopia	tomato	dark green	brown	round	thick	flat
*M. robertsii*	RST-12	Rh. soil	Guder, Ethiopia	tomato	light green	brown	medium round	thick	flat
*M. robertsii*	S-26	soil	Ambo ARC collection	unknown	light green	brown	round	thin, cottony	flat
*M. robertsii*	K-61	Rh. soil	Koka, Ethiopia	tomato	dark green	brown	round	thin, adpressed	flat
*M. robertsii*	K-63	Rh. soil	Koka, Ethiopia	tomato	dark green	brown	round	thin, adpressed	flat
*M. robertsii*	K-101	Rh. soil	Koka, Ethiopia	tomato	light green	brown	medium round	thick, cottony	slightly raised
*M. robertsii*	*K*-7	Rh. soil	Koka, Ethiopia	tomato	light green	brown	round	thin, adpressed	slightly raised
*M. robertsii*	K-102	Rh. soil	Koka, Ethiopia	tomato	light green	dark brown	medium round	thick, cottony	slightly raised

Note: Rh=Rhizosphere, Ambo ARC=Ambo Agriculture Research Center.

**Figure 1.**
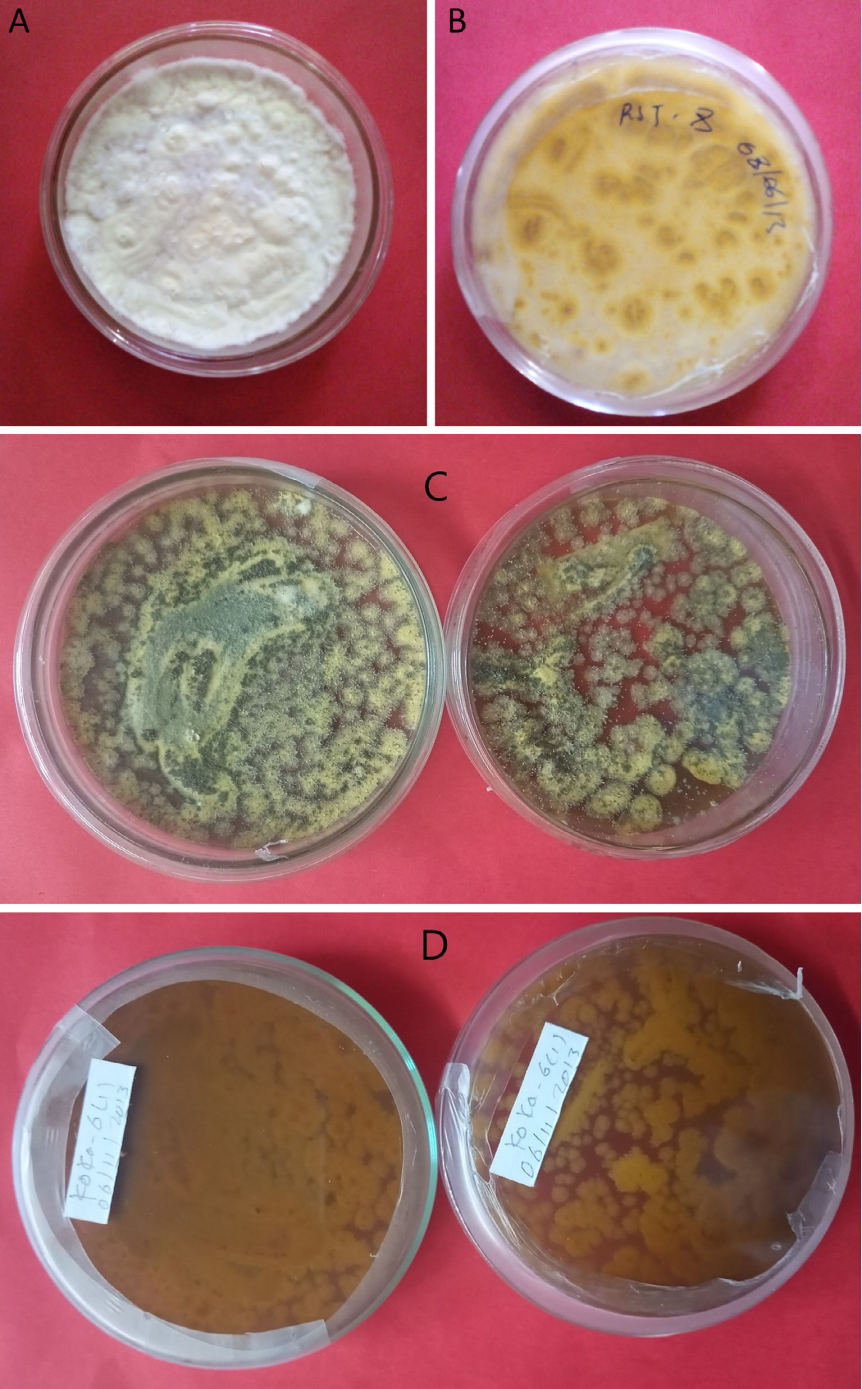
Morphology of *B. bassiana* and *M. robertsii* isolates. (A) Colony color (front) and (B) colony color (back) of *B. bassiana.* (C) Colony color (front) and (D) colony color (back) of *M. robertsii.* No manipulations were made to the image.

*Metarhizium robertsii* isolates showed dark green to light green colony color at the front and brown at the backside, medium round to round colony shape, and flat to slightly raised colony elevation. Isolates K-101, K-102, RST-12, and S-26 showed a light green colony color, whereas K-61 and K-63 showed a dark green ( Figure 1). The surface texture was thin, thick, and cottony.

**Figure 2.**
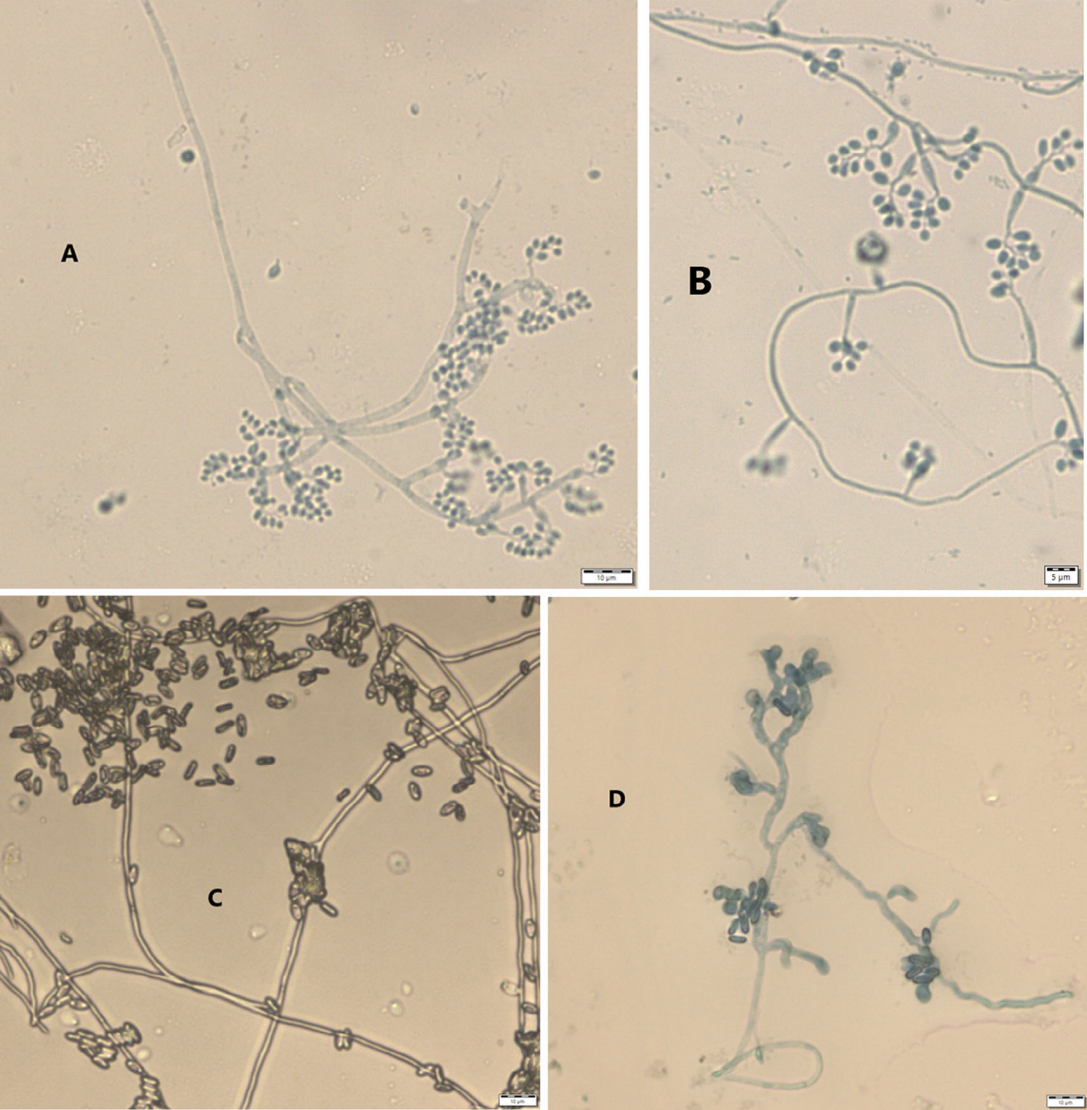
Microscopic feature of *B. bassiana* and *M. robertsii* isolates. (A and B) *B. bassiana.* (C and D) *M. robertsii.* No manipulations were made to the images.

**Figure 3.**
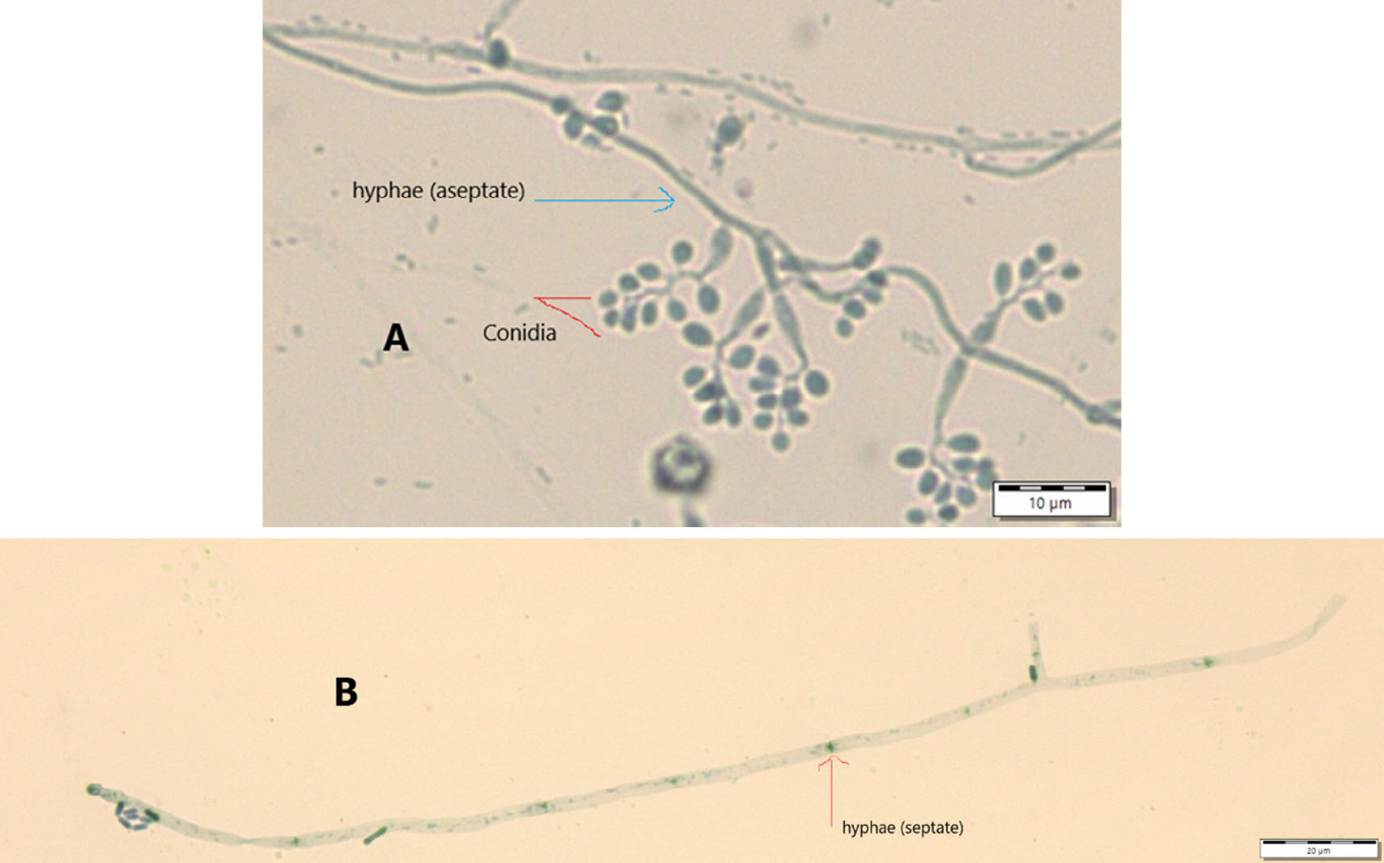
Conidial structure and hyphae of *B. bassiana* and *M. robertsii* isolates. A= *B. bassiana*, B= *M. robertsii*. No manipulations were made to the images.

Results from microscopic features indicated that *B. bassiana* isolates showed a flask-like shape in a conidiogenous cell, a branched conidiophore, and hyaline and smooth conidia with a spherical to subglobose arrangement ( Figure 2) and aseptate hyphae ( Figure 3),

*M. robertsii* isolates revealed cylindrical in spore shape, branched, and densely intertwined conidiophore ( Figure 2) and septate hyphal structures ( Figure 3).

### Spore viability

Spore germination was ranging from 79.33 to 99.03% within 18 hours of incubation at 27±1°C ( Table 2). The results showed that there was a statistically highly significant difference among isolates in the percentage of mean germination (F=54.6; DF=14; P<0.0001). Isolate K-61 showed a maximum mean percentage of germination, whereas the minimum mean percentage was recorded by isolate RST-12.

**Table 2.** Germination percentage of *B. bassiana* and *M. robertsii* isolates.

Isolates	Germination % ± SE
*Beauveria bassiana* RST-8	97.13±1.95 ^ab^
*Beauveria bassiana* APPRC-27	98.1±0.59 ^ab^
*Metarhizium robertsii K*-61	99.03±0.78 ^a^
*Metarhizium robertsii K*-102	97.56±1.36 ^ab^
*Metarhizium robertsii* RST-11	95.40±2.91 ^abc^
*Metarhizium robertsii K*-63	97.63±1.82 ^ab^
*Beauveria bassiana* K-5	97.03±1.63 ^ab^
*Beauveria bassiana* APPRC-44BC	95.86±1.07 ^abc^
*Beauveria bassiana* K-91	96.40±1.53 ^abc^
*Metarhizium robertsii K*-101	97.67±1.24 ^ab^
*Metarhizium robertsii K*-7	88.36±4.85 ^abc^
*Metarhizium robertsii* S-26	80.80±9.83 ^bc^
*Beauveria bassiana* APPRC-44BC-1	87.60±5.03 ^abc^
*Metarhizium robertsii* RST-12	79.33±3.84 ^c^
Tween 80 (Control)	0.00±0.00 ^d^
HSD (0.05)	17.7
CV	6.71

Note: Means with the same letter, in the same column, are not significantly different according to Tukey’s studentized range (HSD) test at, α=0.05. CV, Coefficient of Variance.

### Conidial yield and size

Statistical analysis showed a highly significant difference among isolates at P<0.0001 in their conidial production. Conidial production varies from 0.11 × 10 ^7^ to 2.67 × 10 ^7^ spores ml ^-1^. Maximum spore production was attained by isolate APPRC-27, followed by isolate K-61, whereas the minimum conidial yield was recorded by isolate APPRC-44BC ( Table 3). The length of the conidia showed a mean value of 2.08 to 3.24 μm for *B. bassiana* isolates, and a higher conidial length was achieved by isolate APPRC-27 with 3.24 μm. The highest and lowest conidial diameters were recorded by isolates APPRC-27 and K-91, respectively.

**Table 3.** Conidial yield and size of isolates.

Isolate	Conidial yield/ml ± SE	Conidial length (μm) ± SE	Conidial width (μm) ± SE	Length/width ratio ± SE
*B. bassiana* APPRC-44BC	0.11 × 10 ^7^±0.16 ^e^	2.85±0.03 ^cd^	1.86±0.16 ^c^	1.56±0.12 ^c^
*M. robertsii* K-63	1.67 × 10 ^7^±0.02 ^cb^	7.10±0.24 ^a^	2.49±0.06 ^a^	2.86±0.13 ^ab^
*B. bassiana* K-5	0.17 × 10 ^7^±0.01 ^e^	2.23±0.02 ^d^	1.45±0.05 ^d^	1.54±0.07 ^c^
*M. robertsii* K-102	1.81 × 10 ^7^±0.18 ^cb^	6.43±0.11 ^ab^	1.93±0.03 ^c^	3.32±0.03 ^a^
*B. bassiana* APPRC-27	2.67 × 10 ^7^±0.17 ^a^	3.24±0.11 ^c^	1.99±0.06 ^c^	1.63±0.05 ^c^
*B. bassiana* K-91	1.53 × 10 ^7^±0.01 ^cd^	2.08±0.07 ^d^	1.36±0.03 ^d^	1.53±0.03 ^c^
*B. bassiana* RST-8	1.28 × 10 ^7^±0.04 ^d^	2.51±0.05 ^cd^	1.64±0.11 ^cd^	1.54 ^c^
*M. robertsii* K-61	1.85 × 10 ^7^±0.15 ^b^	5.78±0.48 ^b^	2.36±0.04 ^ab^	2.44±0.20 ^b^
HSD at (0.05)	0.29	0.99	0.40	0.54
CV (%)	7.46	10.40	8.93	11.15

Note: Means with the same letter, in the same column, are not significantly different according to Tukey’s Studentized Range (HSD) test at, α=0.05.

Regarding *M. robertsii* isolates, maximum conidial length and diameter were registered by isolate K-63, while minimum conidial length was obtained by isolate K-61 ( Table 3). The lowest conidial diameter was recorded by isolate K-102 (1.93 μm) when compared to others ( Figure 3).

### Comparison of colony radial growth on a different media

**Figure 4.**
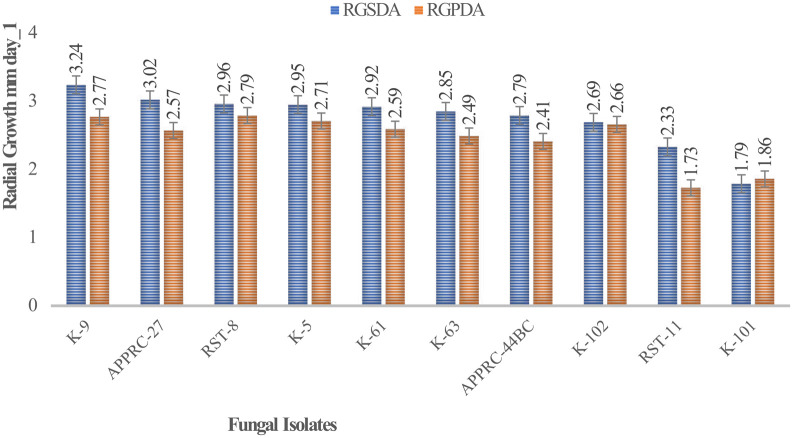
Mean radial growth of isolates on SDA and PDA media. SDA, Sabouraud Dextrose Agar; PDA, Potato Dextrose Agar; RGSDA, radial growth on SDA; RGPDA, radial growth on PDA. No manipulations were made to the images.

Colony radial growth rates ranged from 1.73 to 3.24 mm day ^-1^ ( Figure 4). The highest colony radial growth rate was achieved by *B. bassiana* isolate K-91 on SDA, which increased by 16.96% radial growth compared to PDA media. The minimum radial growth rate was scored by isolate 44BC on both media.

Concerning *M. robertsii* isolates, K-61 showed the highest radial growth with 2.92 on SDA media, followed by K-63 (2.85) mm day ^-1^. Isolate RST-11 showed the lowest colony growth on PDA media.

In this study, the maximum growth rate was recorded on SDA media rather than PDA after 15 days of incubation at 27°C±1 for both species of isolates.

### Pathogenicity screening of isolates on *G. mellonella*

Most isolates were virulent to Great Wax Moth ( *G. melonella **)*** of second instar larvae, with 75–100% mortality after seven days of inoculation of treatments ( Table 4). The mean percentage of mortality of *G. melonella* larvae by *B. bassiana* and *M. robertsii* was highly variable (F = 101.49; DF = 10; P < 0.0001). Maximum mean percentage mortality was achieved by *M. robertsii* isolates K-61 and K-102, while minimum mortality was recorded by *M. robertsii* isolate RST-11.

**Table 4.** Screening of *B. bassiana* and *M. robertsii* isolates on *G. melonella*.

Fungal isolates	Percentage of mortality ± SE
*Metarhizium robertsii* K-61	100±0.00 ^a^
*Metarhizium robertsii* K-102	100±0.00 ^a^
*Metarhizium robertsii* K-63	97.5±0.25 ^ab^
*Beauveria bassiana* K-91	95.00±0.28 ^abc^
*Beauveria bassiana* APPRC-27	92.5±0.25 ^abcd^
*Beauveria bassiana* RST-8	90.00±0.28 ^abcd^
*Beauveria bassiana* APPRC-44BC	85.00±0.28 ^bcde^
*Beauveria bassiana* K-5	82.5±0.47 ^cde^
*Metarhizium robertsii* K-101	80.00±0.40 ^ed^
*Metarhizium robertsii* RST-11	75.00±0.28 ^e^
Control (Tween 80)	7.5±0.25 ^f^
HSD at (0.05)	1.37
CV (%)	6.31

Note: Means with the same letter, in the same column, are not significantly different according to Tukey’s Studentized Range (HSD) test at, α=0.05.

## Discussion

The collected data from the rhizosphere soil of tomatoes above 70% belongs to the typical characteristics of *M. robertsii.* In the present study, six *B. bassiana* and eight *M. robertsii* were morphologically characterized to understand the potential virulence of entomopathogenic fungi. *B. bassiana* has a white colony color and is round in shape, whereas *M. robertsii* has a dark to light colony color and is medium-round to round in shape. Similar work was done by Deb and Dutta (2021). This result was confirmed by the previous findings of different researchers, they reported *M. robertsii* isolates indicating a round colony shape, thin to thick adpressed texture, and flat to slightly raised elevation ( Sepúlveda *et al*., 2016).

This study also detected a microscopic feature of isolates. Consequently, *B. bassiana* had aseptate hyphae and *M. robertsii* showed a cylindrical shape with septate hyphae. A similar study reported that microscopic characterization of *B. bassiana* isolates indicated round to slightly round conidial shape with glassy-like aseptate hyphae, while *M. robertsii* were described as having cylindrical conidial shape, septate hyphae, and branched conidiophores in the form of a candelabrum ( Bich *et al*., 2021).

Germination is one component that affects the degree of virulence; the faster the germination rate, the higher the fungal infection of pests ( Herlinda, 2010). This study showed that the highest conidial germination was achieved by *M. robertsii* isolate K-61 (99.03%) within 18 hours of incubation. This result is consistent with a study conducted by Gebremariam *et al*., (2021), in which spore germination percentage varied between 85.43 and 99.67% within 24 hours of incubation for 26 isolates. Likewise, conidial germination of *Beauveria* isolates ranged from 76.33 to 95.75% ( Belay and Tenkegna, 2017), and 89.30 to 99% of spore viability were recorded by 22 isolates of *B. bassiana* and *M. robertsii* ( Mkiga *et al*., 2020).

Maximum sporulation rate, radial growth, and fast germination rate were the most important parameters for determining virulent isolates of entomopathogenic fungi, and the variation in virulence among isolates may be due to the geographical source of isolates and the potential of fungal species ( Dotaona *et al*., 2015; Gebremariam *et al.,* 2021). This result revealed that the highest radial growth rates were obtained by *B. bassiana* isolate K-91 (3.24 mm/day) and *M. robertsii* isolate K-61 (2.92 mm day ^-1^) on SDA media at 27°C ±. This is consistent with M. *robertsii* developed on SDA media at 30°C, the temperature at which the rates ranged from 0.14 to 3.39 mm day ^-1^ ( Rodríguez *et al.,* 2009). The present study revealed that all isolates showed maximum colony radial growth on SDA media rather than PDA. This may be due to the variation in nutrient contents between the media as SDA is selective. However, Gebremariam *et al*. (2021) reported that the radial growth of *B. bassiana* and *M. robertsii* isolates attained between 0.83 and 3.43-mm day ^-1^ by AAUEM-3 and AAUMFB-77 isolates, respectively, which were grown on PDA media at 25°C.

In the case of *M. robertsii* isolates K-61 and K-102 showed higher virulence levels than other fungal isolates against *G. melonella* under laboratory conditions at seven days post-inoculation. The result confirmed the findings of previous work ( Ibrahim *et al*., 2016). All isolates of *B. bassiana* and *M. robertsii* showed greater than 86% larval mortality, which indicated that isolates were virulent against *G. mellonella.* These findings were agreed upon in the work of Kebede (2019), in which it was reported that the top eight isolates of *B. bassiana* and *M. robertsii* were virulent to *G. mellonella* with above 75% larval mortality.

The results from microscopic features such as conidial length and width revealed that there was a highly significant difference among isolates. Accordingly, the highest conidial length was obtained with 7.10 and 3.24 μm for *M. robertsii* isolate K-63 and *B. bassiana* isolate APPRC-27, respectively ***.*** This agrees with previous work conducted by Doolotkeldieva *et al*. (2019), who reported the conidial length and width of *B. bassiana* were 2.27 and 1.85 μm, respectively, with a length to width ratio of 1.23 μm. Bich *et al*. (2021) also indicated that the conidial diameters of *B. bassiana* showed a mean value of 1.7 to 2.3 μm. Mongkolsamrit *et al.* (2020) also stated that *M. robertsii* showed a cylindrical to ellipsoid conidial shape with 5-7 and 2-3 μm in conidial length and width, respectively. However, Sepúlveda *et al*. (2016) reported that lower conidial lengths vary between 4.52 and 5.54 μm for *M. robertsii* strains. This may be due to the variation in the ecology of fungal species.

## Conclusions

Correct identification of entomopathogenic fungi through morphological characterization is a baseline for the selection of virulent isolates for the management of agricultural pests. There was a variation in morphological features among all isolates in terms of colony color, shape, and texture. Isolates with the highest germination percentage and maximum spore production were virulent to second instar larvae of *G. mellonella.* Accordingly, *M. robertsii* isolates K-61 and K-102 were highly virulent to *G. melonella* by the Galleria dipping method, with a 100% mortality rate at 10 days post inoculation at a concentration of 1 × 10 ^8^ conidial ml ^-1^. This study showed that virulent *B. bassiana* and *M. robertsii* isolates collected from rhizospheric soil had the potential to control pests there is by reducing the use of synthetic pesticides, making them sustainable and cost-effective, and increasing crop production and productivity using this eco-friendly method. Hence, it is recommended to use *B. bassiana* and *M. robertsii* due to their endophytic properties for the management of agricultural pests, particularly insect pests. Moreover, further studies are needed on the factors attributed to the distribution, ecology, efficacy, and root association of *B. bassiana* and *M. robertsii* with tomato and other Solanaceae plants against pests.

## Data Availability

DANS-EASY: Entomopathogenic fungi characterization data, https://doi.org/10.17026/dans-27p-epm2 ( Geremew, 2023). This project contains the following underlying data:
• Germination (germination test of entomopathogenic isolates) • Radial growth PDA (radial growth of isolates on PDA media) • Radial growth SDA (radial growth of isolates on SDA media) • Screening (pathogenicity screening of isolates on *Galleria melonella*) • Conidial length and width (conidial size of isolates) • Conidial yield (conidial yield of isolates) Data are available under the terms of the Creative Commons Zero “No rights reserved” data waiver (CC0 1.0 Public domain dedication).
