# Supplementary material for: Isolation, morphological characterization, and screening virulence of Beauveria bassiana and Metarhizium robertsii fungal isolates  on Galleria mellonella
Source: F1000Res. 2024 Sep 11;12:827. Originally published 2023 Jul 13. [Version 5] doi: 10.12688/f1000research.134020.5 (PMC10905152; doi:10.12688/f1000research.134020.5)
Supplement: Peer review history

# Reviewer report: Reviewer response for version 4

**DOI:** 10.5256/f1000research.161449.r243892

**Published:** 2024-02-28

**Authors:** Linnet Gohole

**Affiliations:** 1 University of Eldoret, Eldoret, Uasin Gishu County, Kenya

Discussion

 1. The first sentence of the discussion is still confusing. I suggest that it should be re-written to read "Of the fungal isolates collected from the soil rhizosphere of tomato plants, 70% of them had characteristics typical of the species M. robertsii". 

 2. The discussion is still largely missing on scientific explanations to the findings obtained. Had this been done, then the manuscript would have been scientifically richer.

Is the work clearly and accurately presented and does it cite the current literature?

Partly

If applicable, is the statistical analysis and its interpretation appropriate?

I cannot comment. A qualified statistician is required.

Are all the source data underlying the results available to ensure full reproducibility?

No source data required

Is the study design appropriate and is the work technically sound?

Yes

Are the conclusions drawn adequately supported by the results?

Partly

Are sufficient details of methods and analysis provided to allow replication by others?

Yes

Reviewer Expertise:

Entomology, biological control of insect pests and integrated pest management.

I confirm that I have read this submission and believe that I have an appropriate level of expertise to confirm that it is of an acceptable scientific standard.

# Response

**Published:** 2024-08-06

**Authors:** Dereje Geremew

**Affiliations:** Crop protection, Ethiopian Institute of Agricultural Research, Ambo, Oromia, Ethiopia

Dear Reviewer,

 All comments are accepted and included in the new version of article.

# Reviewer report: Reviewer response for version 3

**DOI:** 10.5256/f1000research.158147.r219340

**Published:** 2023-11-08

**Authors:** Linnet Gohole

**Affiliations:** 1 University of Eldoret, Eldoret, Uasin Gishu County, Kenya

I have looked at the revised version of the manuscript (Version 3) and going by the comments earlier made bullet by bullet, I note only 3 of the queries have been addressed. The purpose of giving feedback is to improve the manuscript and I strongly recommend that the comments are considered seriously and incorporated accordingly to improve the quality of the paper.

Is the work clearly and accurately presented and does it cite the current literature?

Partly

If applicable, is the statistical analysis and its interpretation appropriate?

I cannot comment. A qualified statistician is required.

Are all the source data underlying the results available to ensure full reproducibility?

No source data required

Is the study design appropriate and is the work technically sound?

Yes

Are the conclusions drawn adequately supported by the results?

Partly

Are sufficient details of methods and analysis provided to allow replication by others?

Yes

Reviewer Expertise:

Entomology, biological control of insect pests and integrated pest management.

I confirm that I have read this submission and believe that I have an appropriate level of expertise to confirm that it is of an acceptable scientific standard, however I have significant reservations, as outlined above.

# Response

**Published:** 2024-01-15

**Authors:** Dereje Geremew

**Affiliations:** Crop protection, Ethiopian Institute of Agricultural Research, Ambo, Oromia, Ethiopia

General comment

 Pathogenicity and virulence are not interchangeable, as the virulence of isolates tested in the experiment. I accepted the comment and modified it accordingly.

# Response

**Published:** 2024-01-15

**Authors:** Dereje Geremew

**Affiliations:** Crop protection, Ethiopian Institute of Agricultural Research, Ambo, Oromia, Ethiopia

All necessary comments were accepted and included in the new version of the article. The title of the article was also modified accordingly.

# Reviewer report: Reviewer response for version 3

**DOI:** 10.5256/f1000research.158147.r219341

**Published:** 2023-10-31

**Authors:** Tinatin Doolotkeldieva

**Affiliations:** 1 Faculty of Agriculture, Kyrgyz-Turkish Manas University, Bishkek, Chuy Province, Kyrgyzstan

The authors have significantly improved the manuscript material; I, as a reviewer, confirm that the published article has undergone adjustments according to the reviewer’s recommendations.

Is the work clearly and accurately presented and does it cite the current literature?

Yes

If applicable, is the statistical analysis and its interpretation appropriate?

Partly

Are all the source data underlying the results available to ensure full reproducibility?

Yes

Is the study design appropriate and is the work technically sound?

Yes

Are the conclusions drawn adequately supported by the results?

Partly

Are sufficient details of methods and analysis provided to allow replication by others?

Partly

Reviewer Expertise:

Biocontrol, entomopthogenic bacteria, fungi, soil microbiology, plant pathogology, biopecticides, bioremediation

I confirm that I have read this submission and believe that I have an appropriate level of expertise to confirm that it is of an acceptable scientific standard.

# Reviewer report: Reviewer response for version 2

**DOI:** 10.5256/f1000research.154480.r198218

**Published:** 2023-09-20

**Authors:** Linnet Gohole

**Affiliations:** 1 University of Eldoret, Eldoret, Uasin Gishu County, Kenya

1. Suggestion for title: Isolation, morphological characterization and screening virulence of *B.* *bassiana* and *M. robertsii* fungal isolates in *G. melonella*.

**Abstract**

 2. Background - the word asymptote should read asymptomatic.

 3. Methods - *Galleria *bait method is used to isolate fungi and not to identify them.

 4. Amended conclusion - what is the rationale for singling out sucking insects as candidate pests to be controlled using the virulent fungal isolates, why not consider insect pests in general? 

**Introduction:**

 5. The following sentence does not convey the intended message "Moreover, entomopathogenic fungi are the immediate solution for pests that feed inside leaves, stems, roots, and seeds because they reduce their exposure to synthetic insecticides and other control methods ( Resquín-Romero *et al*., 2016)."

 6. Was there anything special about collecting the fungal isolates from the rhizosphere of tomato plants?

**Methods**

 7. Under morphological characterization, there is mention of 3 *B. bassiana* and one *M. robertsii* characterized for comparison purposes with new isolates. Can it be clarified why these 4 isolates were included in the study.

 8. How many isolates were eventually tested in the various experiments? The number of treatments keeps changing with each experiment and no explanation is given on how these isolates were selected.

 9. "To hatch third- to fourth-instar larvae, the eggs were laid on folded tissue paper ...." Was this sentence meant to inform readers of the egg laying process, hatching and eventually obtaining 3rd - 4th instar larvae to be used in the pathogenicity experiments? Eventually 2nd instar larvae were used. Clarify the use of the 3rd - 4th instars in the section on rearing Galleria. It is confusing.

 10. Replace the word deepened with dipped. Larvae were dipped in spore suspension. 

 11. Did collection of cadavers begin only after the larvae had been incubated for 10 days? If so, then for how long was this data collected? At what point was the greater than 90% mortality determined? i.e. after how many days? It cannot be indefinite.

**Results**

 12. Fig 2. The hyphae in A look more septate than those in C. Better photos clearly showing the difference need to be taken.

 13. The titles of the tables and figures need to be complete and more informative e.g. Table 4 is about % mortality caused by the various fungal isolates and not screening.

**Discussion**

 14. The first sentence of the discussion is not clear.

 15. The discussion section needs to go beyond repeating the results obtained and whether the agree or disagree with previous findings. It should focus more on scientific explanations of the interesting and unique findings of the current study. e.g. what could be the reason for some fungal isolates being more virulent than others; what do the findings imply when it comes to them being used as biological control agents; is there any connection between the isolate characteristics and virulence with where they were collected or the target crop - tomato?

 16. The isolates were collected from two different locations with different ecological conditions, did this factor have some influence on the isolates collected and their performance?

 17. Some already known isolates were added to the experiments, what does this finally mean?

**Conclusion**

 18. The conclusion on reduction of usage of synthetic pesticides just because the fungal isolates showed high degree of pathogenicity was not a subject of investigation in this study. The conclusions should come directly from the study. Then the implications of these findings can then be speculated and areas of further research recommended.

**General comments**

 1. The authors need to reconstruct some sentences to make them grammatically correct without losing the science being communicated.

 2. Virulence and pathogenicity are used interchangeably in this manuscript. Do they mean the same?

 3. The discussion and conclusion sections should be re-written to capture the suggestions given to help improve the paper.

Is the work clearly and accurately presented and does it cite the current literature?

Partly

If applicable, is the statistical analysis and its interpretation appropriate?

I cannot comment. A qualified statistician is required.

Are all the source data underlying the results available to ensure full reproducibility?

No source data required

Is the study design appropriate and is the work technically sound?

Yes

Are the conclusions drawn adequately supported by the results?

Partly

Are sufficient details of methods and analysis provided to allow replication by others?

Yes

Reviewer Expertise:

Entomology, biological control of insect pests and integrated pest management.

I confirm that I have read this submission and believe that I have an appropriate level of expertise to confirm that it is of an acceptable scientific standard, however I have significant reservations, as outlined above.

# Response

**Published:** 2023-10-18

**Authors:** Dereje Geremew

**Affiliations:** Crop protection, Ethiopian Institute of Agricultural Research, Ambo, Oromia, Ethiopia

Question 9.

 Response: *Galleria melonella* were reared to get a mass of 2nd instar larvae, and 3rd and 4th larvae instar larvae were not used in the experiment.

# Response

**Published:** 2023-10-18

**Authors:** Dereje Geremew

**Affiliations:** Crop protection, Ethiopian Institute of Agricultural Research, Ambo, Oromia, Ethiopia

Question 11. Response

 Cadavers were collected every day after 3 days.

# Response

**Published:** 2023-10-18

**Authors:** Dereje Geremew

**Affiliations:** Crop protection, Ethiopian Institute of Agricultural Research, Ambo, Oromia, Ethiopia

Question 17.

 Response. The performance of some isolates were tested as standard checks with newly collected isolates

# Reviewer report: Reviewer response for version 1

**DOI:** 10.5256/f1000research.147045.r187389

**Published:** 2023-08-09

**Authors:** Tinatin Doolotkeldieva

**Affiliations:** 1 Faculty of Agriculture, Kyrgyz-Turkish Manas University, Bishkek, Chuy Province, Kyrgyzstan

Finding highly virulent entomopathogenic fungi remains an important challenge as an alternative to chemical pesticides. In this article, the authors tried to find two species of entomopathogenic fungi from tomato rhizosphere soil using classical methods of insect pathology, microbiology, entomology, etc.

 However, the authors intended to isolate endophytic fungi, and not from the soil itself; in total, they isolated them from the rhizosphere soil, and not from plant parts.

 Therefore, the authors should realize that they were dealing with soil fungi, this should be emphasized in the conclusions.

 In conclusion, the authors suggest these strains for practical use, but it is too early to speak with such data, after extensive research by testing on other types of sucking insects and for the presence of endophytic properties, it will be possible to recommend them for practical purposes.

Is the work clearly and accurately presented and does it cite the current literature?

Yes

If applicable, is the statistical analysis and its interpretation appropriate?

Partly

Are all the source data underlying the results available to ensure full reproducibility?

Yes

Is the study design appropriate and is the work technically sound?

Yes

Are the conclusions drawn adequately supported by the results?

Partly

Are sufficient details of methods and analysis provided to allow replication by others?

Partly

Reviewer Expertise:

Biocontrol, entomopthogenic bacteria, fungi, soil microbiology, plant pathogology, biopecticides, bioremediation

I confirm that I have read this submission and believe that I have an appropriate level of expertise to confirm that it is of an acceptable scientific standard, however I have significant reservations, as outlined above.

# Response

**Published:** 2023-08-10

**Authors:** Dereje Geremew

**Affiliations:** Crop protection, Ethiopian Institute of Agricultural Research, Ambo, Oromia, Ethiopia

Agreed with the comments, and the comments were included in the conclusion of the article. The conclusion was modified as follows:

 "Correct identification of entomopathogenic fungi through morphological characterization is a baseline for the selection of virulent isolates for the management of agricultural pests. There was a variation in morphological features among all isolates in terms of colony color, shape, and texture. Isolates with the highest germination percentage and maximum spore production were virulent to second instar larvae of *G. mellonella.* Accordingly, *M. robertsii* isolates K-61 and K-102 were highly virulent to *G. melonella* by the Galleria dipping method, with a 100% mortality rate at 10 days post inoculation at a concentration of 1 × 10 ^8^ conidial ml ^-1^. This study showed that virulent *B. bassiana* and *M. robertsii* isolates collected from rhizospheric soil had the potential to control pests by reducing the use of synthetic pesticides, making them sustainable and cost-effective, and increasing crop production and productivity using this eco-friendly method. Hence, it is recommended to use *B. bassiana* and *M. robertsii* due to their endophytic properties for the management of agricultural pests, particularly sucking insects. Moreover, further studies are needed on the factors attributed to the distribution, ecology, efficacy, and root association of *B. bassiana* and *M. robertsii* with tomato and other Solanaceae plants against pests".
